# The efficacy and safety of anti-vascular endothelial growth factor combined with Ahmed glaucoma valve implantation in the treatment of neovascular glaucoma: a systematic review and meta-analysis

**DOI:** 10.3389/fmed.2024.1405261

**Published:** 2024-07-31

**Authors:** Chang-Zhu He, Song-Jie Lu, Zhao-Jun Zeng, Jun-Qiao Liu, Qin Qiu, Fu-Li Xue, Yu He

**Affiliations:** ^1^Chengdu University of Traditional Chinese Medicine, Chengdu, Sichuan, China; ^2^Department of Ophthalmology, Chengdu First People's Hospital/Chengdu Integrated TCM and Western Medicine Hospital, Chengdu, Sichuan, China

**Keywords:** Ahmed glaucoma valve implantation, anti-vascular endothelial growth factor, neovascular glaucoma, meta-analysis, systematic review

## Abstract

**Background:**

The intraocular injections of anti-vascular endothelial growth factor (anti-VEGF) demonstrates significant efficacy in inhibiting the formation of ocular neovascularization in neovascular glaucoma (NVG). Ahmed glaucoma valve implantation (AGVI) is extensively employed for the management of diverse glaucoma types.

**Objective:**

To further evaluate the efficacy and safety of anti-VEGF combined with AGVI in the treatment of neovascular glaucoma.

**Methods:**

A thorough search for randomized controlled trials (RCTs) was conducted across eight databases: PubMed, EMBASE, the Cochrane Library, Web of Science, China National Knowledge Infrastructure, Wanfang, SinoMed, and VIP. The search period was set from the inception of each database until March 2, 2024, to identify RCTs investigating the effectiveness and safety of combining AGVI with anti-VEGF therapy for NVG. We used the Cochrane Risk of Bias Assessment Tool to evaluate the quality of the literature and performed statistical analysis using Stata 15.0 software.

**Results:**

Fourteen RCTs were included in this study. Compared with AGVI alone, the combination of anti-VEGF drugs and AGVI can reduce postoperative intraocular pressure (IOP) at 1 week [WMD = −4.03, 95% CI (−5.73, −2.34), *p* < 0.001], 1 month [WMD = −5.39, 95% CI (−7.05, −3.74), *p* < 0.001], 3 months [WMD = −6.59, 95% CI (−7.85, −5.32), *p* < 0.001], 6 months [WMD = −4.99, 95% CI (−9.56, −0.43), *p* = 0.032], and more than 12 months [WMD = −3.86, 95% CI (−6.82, −0.90), *p* = 0.011], with a higher Effective rate [RR = 1.27, 95% CI (1.18, 1.37), *p* < 0.001], decreased incidence of postoperative hyphema [RR = 0.24, 95% CI (0.15, 0.39), *p* < 0.001], reduced use of postoperative antiglaucoma medications [WMD = −0.48, 95% CI (−0.61, −0.35), *p* < 0.001], and decreased aqueous humor VEGF levels [SMD = −2.84, 95% CI (−4.37, −1.31), *p* < 0.001].

**Conclusion:**

In comparison to AGVI alone, the combination of AGVI with anti-VEGF therapy has better effects in reducing IOP at various time intervals, diminishing postoperative antiglaucoma medication requirements and reducing aqueous humor VEGF levels. Furthermore, it effectively minimizes the incidence of postoperative hyphema. Nevertheless, due to the variability in the quality of the trials included, further high-quality experiments will be required in the future to substantiate this conclusion.

**Systematic review registration:**

PROSPERO, identifier CRD42024519862, https://www.crd.york.ac.uk/prospero/display_record.php?ID=CRD42024519862.

## 1 Introduction

Neovascular glaucoma (NVG) constitutes a form of secondary glaucoma characterized by the emergence of rubeosis iridis and elevated intraocular pressure (IOP), which poses a potential threat to vision impairment (1). Chronically red and painful eyes are the first visible clinical symptoms of NVG, however, in younger patients, these symptoms may be absent owing to insufficient endothelial functional reserve (2). More importantly, NVG typically represents an end-stage disease associated with the potential for blindness, persistent pain, and loss of the eyeball (3). The primary etiologies of NVG include diabetic retinopathy (DR), ischemic central retinal vein occlusion (CRVO), and ocular ischemic syndrome (OIS) (4). According to relevant reports, about 40 to 45% of eyes affected by ischemic retinal vein occlusion are predisposed to developing NVG, with 80% of these cases manifesting within a timeframe of 6 to 8 months (5). Retinal ischemia leads to the release of vascular endothelial growth factors (VEGF), which diffuse into the aqueous humor and anterior segment, triggering the formation of neovascularization in the iris and anterior chamber angle, this process impedes the outflow of aqueous humor, leading to elevated IOP (6). Studies reveal a significant increase in VEGF levels in the aqueous humor of NVG patients, suggesting that VEGF plays a crucial role in mediating active intraocular neovascularization in patients with ischemic retinal diseases (7). Based on data from the European Union, the prevalence of NVG in Europe ranges from 75,000 to 113,000 individuals, constituting 3.9% of the total glaucoma cases (8). In the United States, approximately 17,500 diabetic patients suffer from iris neovascularization, with the majority experiencing proliferative diabetic retinopathy, and the incidence of iris neovascularization is as high as 65% in these patients (9).

The Ahmed valve features a unidirectional pressure-sensitive control mechanism, limiting drainage device operation to IOP levels between 8 and 14 mmHg, thereby mitigating excessive postoperative aqueous humor drainage (10). Therefore, compared to traditional trabeculectomy, the Ahmed valve is more suitable for refractory glaucoma (11). Unfortunately, research reports indicate that AGVI is associated with a high rate of encapsulation and inadequate intraocular pressure reduction (IOPR), necessitating ongoing glaucoma medication postoperatively (12). Anti-VEGF drugs such as ranibizumab and bevacizumab have been demonstrated to efficiently reduce neovascularization progression and leakage in ocular neovascular disease (13). Relevant studies also indicate that supplementary anti-VEGF therapy could be advantageous for neovascular glaucoma, given its anti-angiogenic properties (14).

Among patients with NVG undergoing AGVI, there is still a lack of consensus on the necessity of intraocular injections of anti-VEGF. Several retrospective studies have found that combined AGVI with anti-VEGF drugs can lower postoperative IOP compared to AGVI alone, reduce the use of postoperative antiglaucoma medications, and decrease the occurrence of adverse events (15, 16). Nevertheless, comparative studies by Tang et al. (17) and Ma et al. (18) found that the use of anti-VEGF drugs did not affect the final outcome of AGVI. Currently, there have been several randomized controlled trials (RCTs) comparing the postoperative outcomes of AGVI alone versus AGVI combined with intraocular injections of anti-VEGF for NVG, however, few systematic reviews or meta-analyses have been performed to compare their clinical effects and safety and most of the relevant meta-analyses mainly focus on retrospective studies. Additionally, existing meta-analyses have not examined the different anti-VEGF drugs, nor have they addressed the long-term effects of combining anti-VEGF drugs with AGVI. This study separately discussed three common anti-VEGF drugs (ranibizumab, bevacizumab, and conbercept) and examined their effects when combined with AGVI at intervals of 1 week, 1 month, 3 months, 6 months, and beyond 12 months. Subgroup analysis was also performed on follow-up time to investigate the long-term efficacy of VEGF drugs combined with AGVI. To update the existing data and better evaluate the efficacy and safety of AGVI combined with intraocular injections of anti-VEGF for NVG, we conducted this systematic review to compare the postoperative IOP, effectiveness, number of postoperative anti-glaucoma medications used, the incidence of postoperative hyphema and aqueous humor VEGF levels between the two groups. The objective is to aid clinical ophthalmologists in choosing more appropriate treatment for patients with NVG.

## 2 Methods

The study was registered with PROSPERO (registration number: CRD42024519862) and followed the PRISMA (Preferred Reporting Items for Systematic Reviews and Meta-Analyses) guidelines, consistent with the recommendations of the Cochrane Collaboration (19, 20). Details of the PRISMA checklist can be found in Supplementary materials S1.

### 2.1 Search strategy

PubMed, EMBASE, the Cochrane Library, Web of Science, China National Knowledge Infrastructure, Wanfang Database, SinoMed, and the VIP Database were systematically searched for eligible studies from their inception up to March 2, 2024, by two independent authors (CZH and SJL). The search was conducted without any restrictions based on race, age, or language. We employed medical subject headings (MeSH) along with free terms and a set of keywords to formulate search strategies. For example, when searching English databases, we selected the following five core components: (1) Glaucoma Drainage Implants (e.g., Aqueous Humor Shunt, Shunt, Aqueous Humor, Shunts, Aqueous Humor, Glaucoma Filtration Implant, Aqueous Shunt, Shunt, Aqueous Shunts, Aqueous, Aqueous Humor Shunts, Aqueous Shunts, Glaucoma Drainage Implant, Drainage Implant, Glaucoma, Drainage Implants, Glaucoma, Implant, Glaucoma Drainage, Implants, Glaucoma Drainage, Glaucoma Filtration Implants, Filtration Implant, Glaucoma, Filtration Implants, Glaucoma, Implant, Glaucoma Filtration, Implants, Glaucoma Filtration); (2) vascular endothelial growth factor (e.g., VEGFs); (3) Ranibizumab (e.g., Lucentis, RhuFab V2, V2, RhuFab); (4) Bevacizumab (e.g., Avastin); (5) Glaucoma, Neovascular (e.g., Glaucomas, Neovascular, Neovascular Glaucoma, and Neovascular Glaucomas). Additionally, relevant articles from initial search meta-analyses and grey literature were reviewed and included. Detailed retrieval procedures are outlined in Supplementary materials S2.

### 2.2 Inclusion criteria

1. *Type of study*: RCTs of intraocular injections of anti-VEGF combined with AGVI in the treatment of NVG.
2. *Type of participants*: Patients diagnosed as NVG according to any authoritative clinical guidelines, such as Neovascular glaucoma--etipathogeny and diagnosis (21) or Consensus of Chinese experts on the diagnosis and treatment of neovascular glaucoma (22).
3. *Type of interventions and controls*: All patients diagnosed with NVG underwent AGVI treatment. In the experimental group, NVG patients received both AGVI therapy and intraocular injections of anti-VEGF, including ranibizumab, bevacizumab, and conbercept. We did not impose restrictions on the timing of intraocular anti-VEGF injections, whether administered preoperatively or postoperatively, both injections met the inclusion criteria.
4. *Type of outcomes*: Included studies examined at least one of the following outcomes:

**The primary outcome:**

- Intraocular pressure (IOP): The IOP measurements obtained with the Goldmann applanation tonometer (GAT), the non-contact tonometer (NCT), and the rebound tonometer (RBT) all met the inclusion criteria for this study (23). The study selected IOP data at 1 week, 1 month, 3 months, 6 months, and more than 12 months postoperatively for follow-up.
- Incidence of postoperative hyphema: Hyphema, a potential postoperative complication of ophthalmic surgery, was defined as hemorrhage in the anterior chamber sufficient to form a layered clot, even if minimal. Eyes with only suspended red blood cells in the anterior chamber, without forming a layered clot, were not considered to have hyphema (24). The incidence of postoperative hyphema is primarily determined by the proportion of participants experiencing postoperative hyphema to the total number of participants in the experimental or control group.
- Effective rate: The effective rate was determined by the proportion of participants with significantly improved or recovered symptoms relative to the total number in the test or control group.

**The second outcome:**

d Postoperative antiglaucoma medication requirements: The primary outcome measure of this study revolves around quantifying the quantity of antiglaucoma medications required by NVG patients post-surgery.
e Aqueous humor VEGF levels: VEGF stimulated the growth of retinal endothelial cells *in vitro*, as did vitreous fluid containing measurable VEGF (25).

### 2.3 Exclusion criteria

Exclusion criteria comprise the following criteria: (1) The animal experiments, review articles, case reports, meta-analysis, any non-RCTs were ruled out. (2) The NVG patients who did not use Ahmed glaucoma drainage devices and intraocular injection of anti-VEGF would be excluded. (3) RCTs without relevant outcomes or complete data were not available. (4) Patients with concurrent intraocular diseases that could impact surgical outcomes, including congenital vitreoretinopathies and traumatic retinal detachment.

### 2.4 Data extraction

The two authors (CZH and SJL) conducted initial screening by reviewing the titles and abstracts of the literature, adhering to predefined inclusion and exclusion criteria. Subsequently, the final appropriate literature were identified through comprehensive reading of the full texts. The author, year, region, sample, age of the patient, intervention and control, injection dose and outcomes were extracted independently by two authors (CZH and SJL). When consensus could not be solved by discussion, the third author (YH) were consulted. When part of the data was missing from the included literature, the first author or corresponding author was contacted to acquire the necessary information.

### 2.5 Assessment of risk of bias

Two authors (CZH and SJL) independently assessed the risk of bias of the included studies using the Cochrane Risk of Bias Version 2 (RoB 2) assessment tool (26). RoB 2 includes the following six assessment items: (1) the random sequence generation, (2) deviations from the intended interventions, (3) missing outcome data, (4) measurement of the outcome, (5) selection of the reported result, and (6) overall bias. Each section is assessed as “low risk,” “some concern,” or “high risk,” based on the specific circumstances of the article.

### 2.6 Statistical analysis

The study employed Stata15.0 software for conducting a meta-analysis of the data. For continuous variables, we used the standard mean difference (SMD) or weighted mean difference (WMD) and 95% confidence interval for analysis. For binary variables, we used the risk ratio (RR) and 95% confidence interval for analysis. Considering the great differences in research methodologies, basic characteristics of NVG patients, surgical proficiency of the surgeon in each study was inevitable, statistical heterogeneity was disregarded. As a result, a random-effects model was employed for analysis on all data, irrespective of whether I2 was less than 50%. Additionally, for further investigation, we also conducted subgroup analyses on different anti-VEGF drugs. For all statistical procedures, *p* < 0.05 was considered statistically significant.

### 2.7 Sensitivity analysis

To evaluate the robustness of our findings, we conducted a sensitivity analysis, systematically excluding individual studies in sequence. If the exclusion of an article influenced the outcome and reversed the conclusion, we meticulously scrutinized the complete text of the article to ascertain its role as a potential source of heterogeneity. Conversely, this suggested the stability of the study’s findings.

### 2.8 Assessment of reporting bias

When the number of included studies is greater than or equal to 10, a funnel plot is employed. Initially, visual assessment is utilized to evaluate potential publication bias. Based on the generated funnel plot, the Begg’ test or the Egger’ test was both further utilized to examine publication bias.

## 3 Results

### 3.1 Study selection

**Figure 1:**
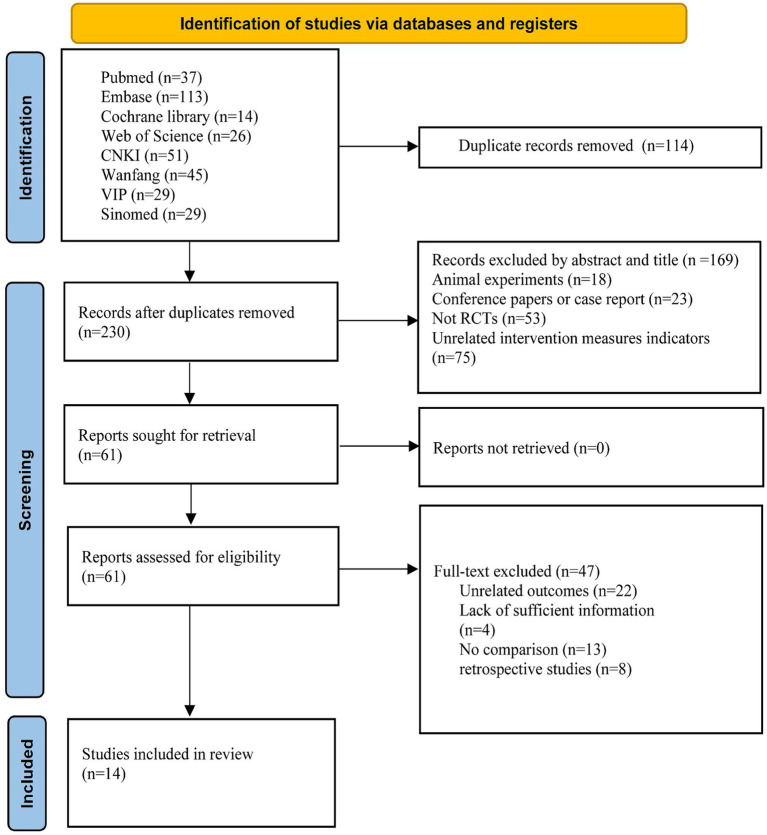
Flow chart of selection studies and specific reasons for exclusion.

Following the initial database search, 344 pieces of literature were retrieved, subsequently, after eliminating duplicate documents, the total has been reduced to 230 pieces. Through reviewing the titles and abstracts, 169 literature pieces were deemed ineligible for inclusion. These exclusions comprised 18 studies involving animal experiments, 23 conference or case reports, 53 non-RCTs, and 75 studies disqualified due to intervention measures not meeting the stipulated criteria. Finally, A total of 14 literature pieces (27–40) met the inclusion criteria. Among the 61 excluded pieces, 22 lacked pertinent outcome measures (including IOP, Incidence of postoperative hyphema, effective rate, postoperative antiglaucoma medication requirements, BCVA and aqueous humor VEGF levels), 13 did not adhere to randomized controlled trial protocols, 8 were retrospective studies and 4 were unable to furnish valid data. The specific literature screening process is shown in Figure 1.

### 3.2 Study characteristics

Among the 14 studies incorporated, one study (27) was from Egypt, one (28) from Iran, one (29) from Brazil, and the remaining studies were all from China. The sample sizes in these studies varied from 9 to 48, spanning the years 2013 to 2022. Moreover, all the studies incorporated were characterized as RCTs. The treatments assessed were as follows: 3 trials (27–29) assessed the effectiveness of AGVI in combination with bevacizumab, while 7 studies (30–36) primarily examined the therapeutic effects of AGVI combined with ranibizumab. Additionally, 3 studies (37–39) concentrated on conbercept, whereas the remaining one did not specify the type of anti-VEGF drug used in the experimental group. Two studies (28, 29) favored postoperative administration, the remaining studies uniformly adopted preoperative administration of anti-VEGF drugs. Three studies (27, 29, 38) explicitly stated that all patients underwent pan-retinal photocoagulation (PRP) therapy, while three others (30, 33, 34) indicated that only a subset of patients received PRP treatment. The remaining studies provided detailed accounts of the utilization of PRP. One study (29) documented three administrations of anti-VEGF drugs, another study (38) utilized them twice, whereas the remaining investigations employed these drugs once. 4 studies (27–29, 33) had a follow-up period of 12 months or more, with the longest extending to 24 months. 3 studies (32, 34, 36) had a 6-month follow-up period, while one study (31) did not report specific follow-up durations. The remaining studies had follow-up periods of 3 months or less, with the shortest being just 1 week. The characteristics of the included studies were detailed in Table 1.

**Table 1:** Basic characteristics of the included studies.

					Age		Intervention regimen		Number of anti-VEGF used		Combine PRP					
References	Year	Region	Study design	Sample(T/C)	T	C	T	C	T	C	T	C	Follow-up	Time of injection	Dosage of anti-VEGF	Outcome
Mahdy RA, et al	2013	Egypt	RCT	20/20	55 ± 1.3	56 ± 4.3	AGVI+B	AGVI	1	1	Yes	Yes	18 months	Pre-operation	1.25 mg/0.05 mL	IOP, Number of hyphaema, Total efficiency
Zarei R, et al	2021	Iran	RCT	30/30	56.7 ± 17.2	57.4 ± 16.4	AGVI+B	AGVI	1	1	NR	NR	12 months	Post-operation	1.25 mg/0.05 mL	IOP, Total efficiency
Arcieri ES	2015	Brazil	RCT	20/20	59.25 ± 8.05	62.40 ± 11.78	AGVI+B	AGVI	3	3	Yes	Yes	24 months	Post-operation	0.05 mL/1.25 mg	IOP, Number of hyphaema, Number of antiglaucoma medications
Xu JH, et al	2015	China	RCT	9/19	NR	NR	AGVI+R	AGVI	1	1	Partial acceptance		1 week	Pre-operation	0.5 mg	IOP, Number of hyphaema
Li YB, et al	2019	China	RCT	46/34	58.06 ± 7.33	57.49 ± 8.42	AGVI+R	AGVI	1	1	NR	NR	NR	Pre-operation	10 mg /ml	Number of hyphaema, Total efficiency
Zong LM, et al	2018	China	RCT	38/35	56.9 ± 8.3	55.2 ± 9.4	AGVI+R	AGVI	1	1	NR	NR	6 months	Pre-operation	1.25 mg/0.05 mL	IOP, Number of hyphaema
Xie Z, et al	2018	China	RCT	31/35	48.32 ± 11.63	52.77 ± 15.22	AGVI+R	AGVI	1	1	Partial acceptance		12 months	Pre-operation	NR	IOP, Number of antiglaucoma medications
Liu H, et al	2019	China	RCT	31/31	55.87 ± 9.84	55.09 ± 10.13	AGVI+R	AGVI	1	1	Partial acceptance		6 months	Pre-operation	1.25 mg/0.05 mL	IOP, Number of hyphaema, VEGF
Xu KK, et al	2022	China	RCT	46/48	36.28 ± 4.37	36.61 ± 3.56	AGVI+R	AGVI	1	1	NR	NR	3 months	Pre-operation	10 mg/mL	Number of hyphaema, Total efficiency, VEGF
Tian LJ, et al	2019	China	RCT	28/24	NR	NR	AGVI+R	AGVI	1	1	NR	NR	6 months	Pre-operation	1.25 mg/0.05 mL	Total efficiency
Liu XR, et al	2020	China	RCT	40/40	49.8 ± 5.7	42.7 ± 5.6	AGVI+C	AGVI	1	1	NR	NR	3 months	Pre-operation	10 mg/mL	IOP, Number of hyphaema, Number of antiglaucoma medications, Total efficiency
Li XY, et al	2022	China	RCT	45/45	50.11 ± 4.2	50.25 ± 4.68	AGVI+C	AGVI	2	2	Yes	Yes	1 months	Pre-operation	NR	IOP, Number of hyphaema, Total efficiency, VEGF
Zheng HF, et al	2021	China	RCT	40/40	52.12 ± 1.03	51.96 ± 1.01	AGVI+C	AGVI	1	1	NR	NR	1 week	Pre-operation	0.5 mg	IOP, VEGF, Total efficiency
Luo GE, et al	2018	China	RCT	28/24	58.8 ± 11.9	58.0 ± 13.3	AGVI+anti-VEGF	AGVI	1	1	NR	NR	1 months	Pre-operation	NR	IOP, Number of hyphaema, Total efficiency, Number of antiglaucoma medications

NR, not report; T, test group; C, control group; RCT, randomized Controlled Trial; AGVI, Ahmed glaucoma valve implantation; IOP, intraocular pressure; VEGF, vascular endothelial growth factor; mg, milligram; ml, milliliter.

### 3.3 Risk of bias

**Figure 2:**
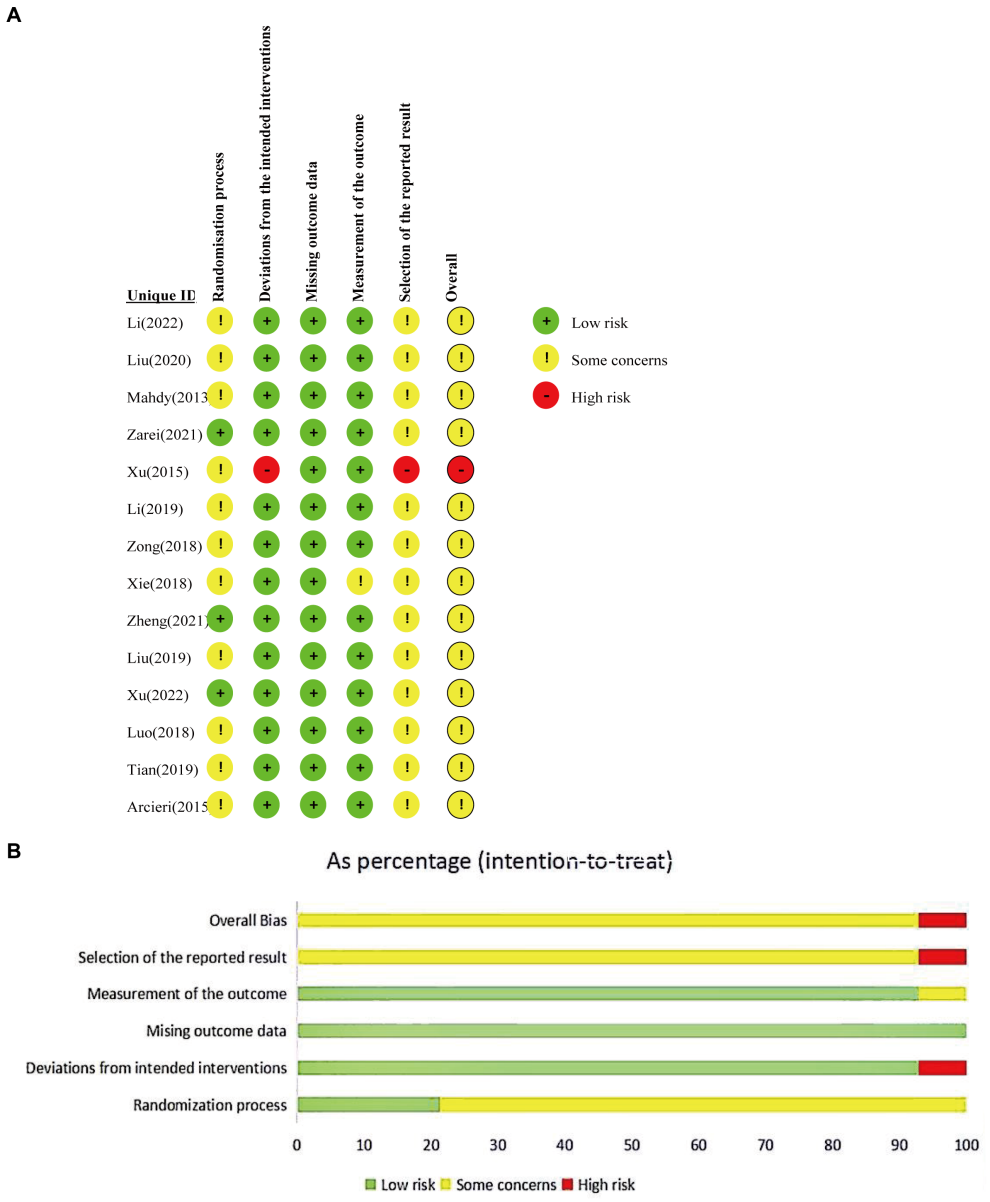
Risk of bias of RCTs. **(A)** Risk of bias graph; **(B)** Risk of bias summary.

The risk of bias in the included RCTs is listed in Figure 2. In the 14 included studies, two (35, 38) utilized the random number table method, while the remaining did not provide specific descriptions of the random allocation method. Only one study (28) explicitly mentioned blinding, while the others were considered to potentially lack blinding, allowing participants and caregivers to know the allocated interventions. One study (28) had missing data, but the data in the other studies were complete, with the missing data falling within an acceptable range. None of the articles deviated from the expected interventions, nor did they selectively report results (Table 2).

**Table 2:** The outcome of the meta-analysis.

Outcomes	Number	Sample size	WMD/RR/SMD	95%CI	Heterogeneity	*p*-value	F/R	Note
IOP at 1 week	6	368	WMD = −4.03	(−5.73, −2.34)	73.20%	*p* < 0.001	R	Overall
	2	108	WMD = −6.24	(−7.35, −5.13)	0%	*p* < 0.001	R	Ranibizumab group
	2	100	WMD = −2.71	(−4.55, −0.87)	10%	*p* = 0.004	R	Bevacizumab group
	2	160	WMD = −3.20	(−5.07, −1.33)	31.90%	*p* = 0.001	R	Conbercept group
IOP at 1 month	6	401	WMD = −5.39	(−7.05, −3.74)	80%	*p* < 0.001	R	Overall
	2	139	WMD = −5.84	(−7.68, −4.01)	0%	*p* < 0.001	R	Ranibizumab group
	2	170	WMD = −4.14	(−6.69, −1.59)	82.10%	*p* = 0.001	R	Conbercept group
IOP at 3 months	5	347	WMD = −6.59	(−7.85, −5.32)	61.80%	*p* < 0.001	R	Overall
	2	167	WMD = −6.83	(−8.06, −5.61)	0%	*p* < 0.001	R	Ranibizumab group
	2	100	WMD = −5.88	(−10.38, −1.39)	89.20%	*p* = 0.010	R	Bevacizumab group
IOP at 6 months	5	301	WMD = −4.99	(−9.56, −0.43)	95.60%	*p* = 0.032	R	Overall
	3	201	WMD = −4.19	(−5.77, −2.62)	15.80%	*p* < 0.001	R	Ranibizumab group
	2	100	WMD = −6.36	(−17.44, 4.71)	98.70%	*p* = 0.260	R	Bevacizumab group
IOP more than 12 months	4	209	WMD = −3.86	(−6.82, −0.90)	86%	*p* = 0.011	R	Overall
Effective rate	9	628	RR = 1.27	(1.18, 1.37)	1.80%	*p* < 0.001	R	Overall
	4	306	RR = 1.27	(1.13, 1.42)	0%	*p* < 0.001	R	Ranibizumab group
	2	170	RR = 1.30	(1.05, 1.62)	49.80%	*p* = 0.017	R	Conbercept group
	2	100	RR = 1.42	(0.88, 2.28)	75.20%	*p* = 0.149	R	Bevacizumab group
Incidence of postoperative hyphema	10	641	RR = 0.24	(0.15, 0.39)	0%	*p* < 0.001	R	Overall
	5	337	RR = 0.27	(0.13, 0.57)	0%	*p* = 0.001	R	Ranibizumab group
	2	100	RR = 0.26	(0.12, 0.56)	0%	*p* = 0.001	R	Bevacizumab group
	2	170	RR = 0.34	(0.07, 1.68)	0%	*p* = 0.186	R	Conbercept group
Aqueous humor VEGF levels	4	326	SMD = −2.84	(−4.37, −1.31)	95.80%	*p* < 0.001	R	Overall
Postoperative antiglaucoma medication requirements	4	238	WMD = −0.49	(−0.62, −0.37)	0%	*p* < 0.001	R	Overall

WMD, weighted mean difference; SMD, standard mean difference; RR, risk ratio; CI, confidence interval; *p*, *p* value represents clinical significance; T, test group; C, control group; F, fixed effects model; R, random effects model; IOP, intraocular pressure; VEGF, vascular endothelial growth factor.

### 3.4 Effects of interventions

#### 3.4.1 IOP at 1 week postoperatively

**Figure 3:**
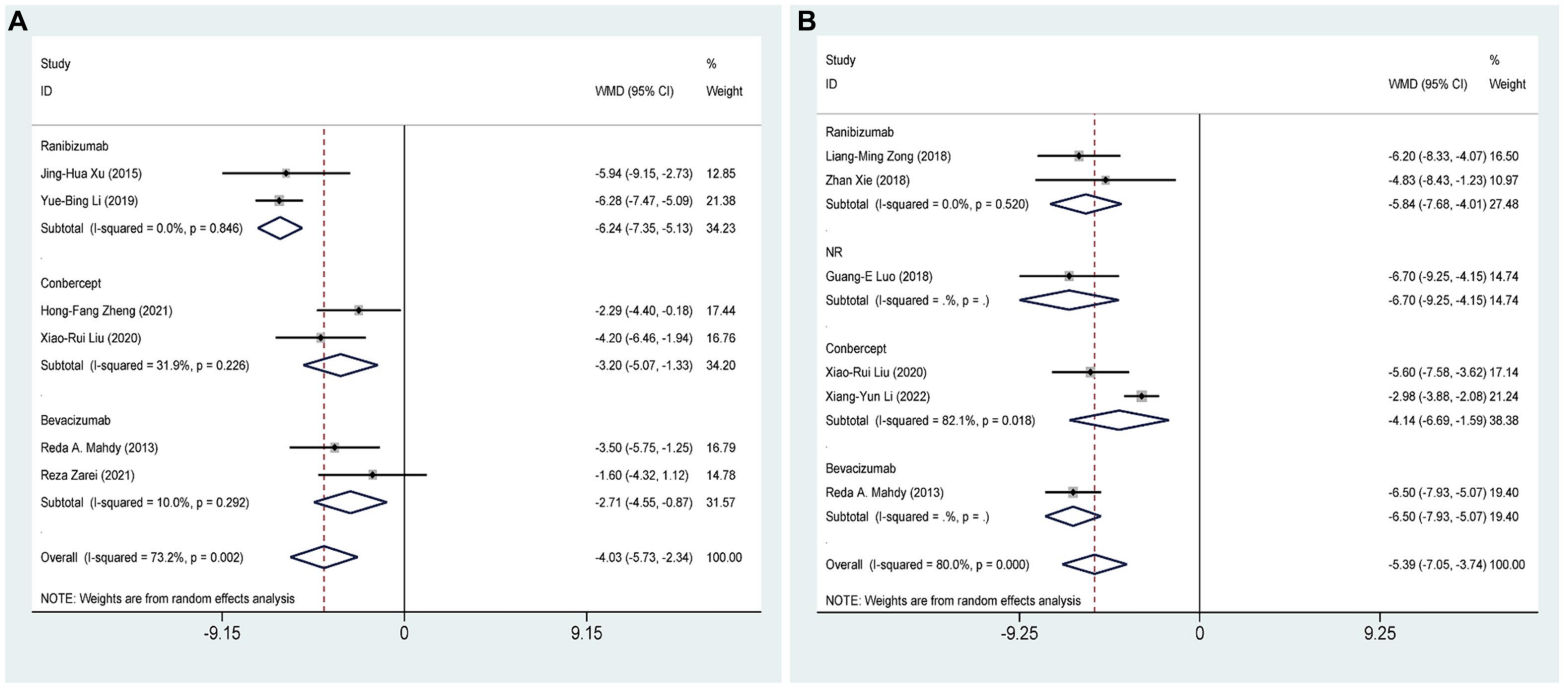
AGVI alone vs. AGVI combined with anti-VEGF drugs: **(A)** IOP at 1 week: subgroup analysis based on anti-VEGF drugs; **(B)** IOP at 1 month: subgroup analysis based on anti-VEGF drugs.

Six RCTs (27, 28, 30, 31, 37, 39), comprising 368 eyes, investigated the effects of combined AGVI with anti-VEGF drugs versus AGVI alone on IOP at 1 week postoperatively. Pooled results showed that AGVI combined with anti-VEGF was more effective in lowering IOP in NVG patients 1 week after surgery compared to AGVI alone [WMD = −4.03, 95% CI (−5.73, −2.34), *p* < 0.001]. The results of subgroup analysis indicated that the ranibizumab group, bevacizumab group and conbercept group all demonstrated superior efficacy in reducing intraocular pressure at 1 week postoperatively compared to AGVI alone ([WMD = −6.24, 95% CI (−7.35, −5.13), *p* < 0.001], [WMD = −2.71, 95% CI (−4.55, −0.87), *p* = 0.004] and [WMD = −3.20, 95% CI (−5.07, −1.33), *p* = 0.001] respectively) (Figure 3A).

#### 3.4.2 IOP at 1 month postoperatively

Six RCTs (27, 32, 33, 37, 38, 40), comprising 401 eyes, investigated the effects of combined AGVI with anti-VEGF drugs versus AGVI alone on IOP at 1 month postoperatively. Pooled results showed that AGVI combined with anti-VEGF was more effective in lowering IOP in NVG patients 1 month after surgery compared to AGVI alone [WMD = −5.39, 95% CI (−7.05, −3.74), *p* < 0.001]. The results of subgroup analysis indicated that the ranibizumab group and conbercept group all demonstrated superior efficacy in reducing intraocular pressure at 1 month postoperatively compared to AGVI alone ([WMD = −5.84, 95% CI (−7.68, −4.01), *p* < 0.001] and [WMD = −4.14, 95% CI (−6.69, −1.59), *p* = 0.001] respectively) (Figure 3B).

#### 3.4.3 IOP at 3 months postoperatively

**Figure 4:**
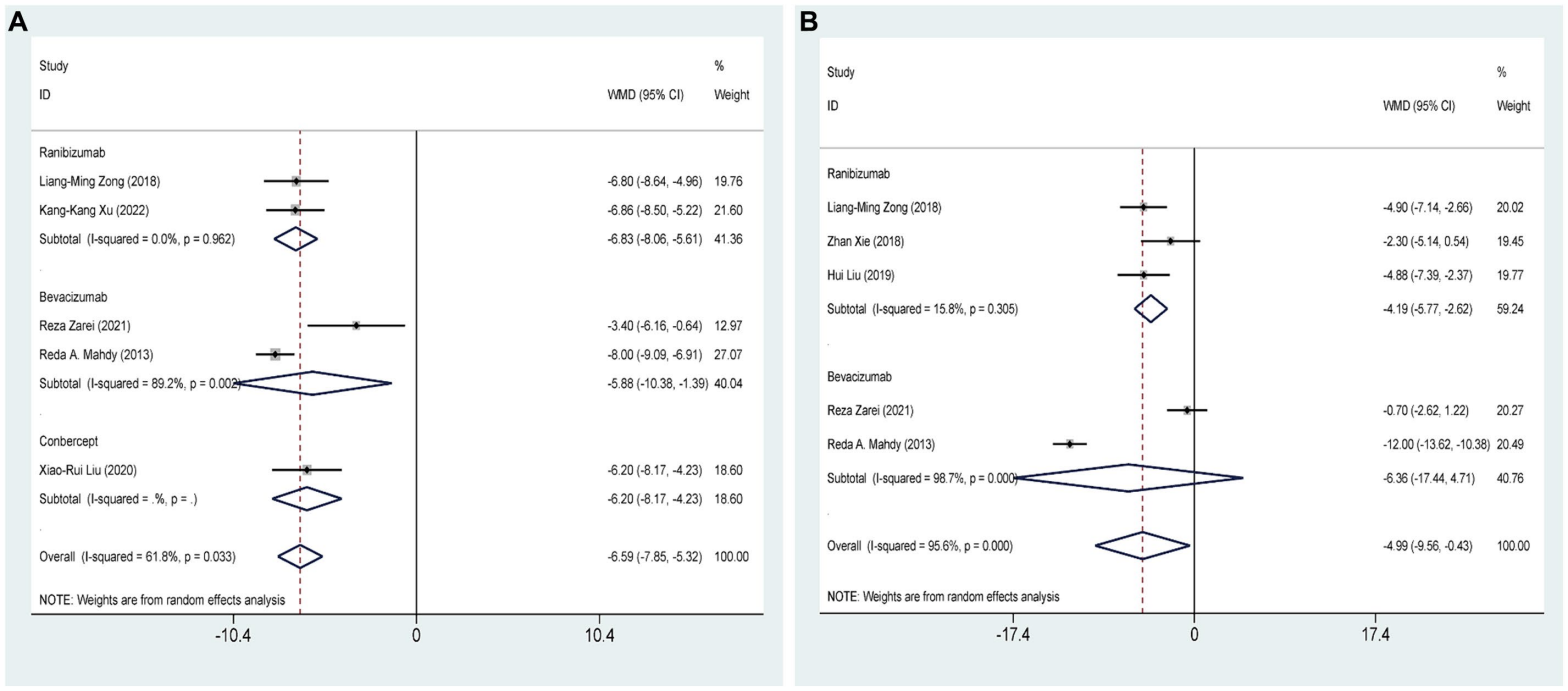
AGVI alone vs. AGVI combined with anti-VEGF drugs: **(A)** IOP at 3 months: subgroup analysis based on anti-VEGF drugs; **(B)** IOP at 6 months: subgroup analysis based on anti-VEGF drugs.

Five RCTs (27, 28, 32, 35, 37), comprising 347 eyes, investigated the effects of combined AGVI with anti-VEGF drugs versus AGVI alone on IOP at 3 months postoperatively. Pooled results showed that AGVI combined with anti-VEGF was more effective in lowering IOP in NVG patients 3 months after surgery compared to AGVI alone [WMD = −6.59, 95% CI (−7.85, −5.32), *p* < 0.001]. The results of subgroup analysis indicated that the ranibizumab group and bevacizumab group all demonstrated superior efficacy in reducing intraocular pressure at 3 months postoperatively compared to AGVI alone ([WMD = −6.83, 95% CI (−8.06, −5.61), *p* < 0.001] and [WMD = −5.88, 95% CI (−10.38, −1.39), *p* = 0.010] respectively) (Figure 4A).

#### 3.4.4 IOP at 6 months postoperatively

Five RCTs (27, 28, 32–34), comprising 301 eyes, investigated the effects of combined AGVI with anti-VEGF drugs versus AGVI alone on IOP at 6 months postoperatively. Pooled results showed that AGVI combined with anti-VEGF was more effective in lowering IOP in NVG patients 6 months after surgery compared to AGVI alone [WMD = −4.99, 95% CI (−9.56, −0.43), *p* = 0.032]. The results of subgroup analysis indicated that the ranibizumab group demonstrated superior efficacy in reducing intraocular pressure at 6 months postoperatively compared to AGVI alone [WMD = −4.19, 95% CI (−5.77, −2.62), *p* < 0.001]. Nevertheless, it is important to mention that the outcomes of bevacizumab group did not reach statistical significance (*p* = 0.260) (Figure 4B).

#### 3.4.5 IOP more than 12 months postoperatively

Four RCTs (27–29, 33), comprising 209 eyes, investigated the effects of combined AGVI with anti-VEGF drugs versus AGVI alone on IOP more than 12 months postoperatively. Pooled results showed that AGVI combined with anti-VEGF was more effective in lowering IOP in NVG patients more than 12 months after surgery compared to AGVI alone [WMD = −3.86, 95% CI (−6.82, −0.90), *p* = 0.011] (Supplementary materials S3).

#### 3.4.6 Effective rate

**Figure 5:**
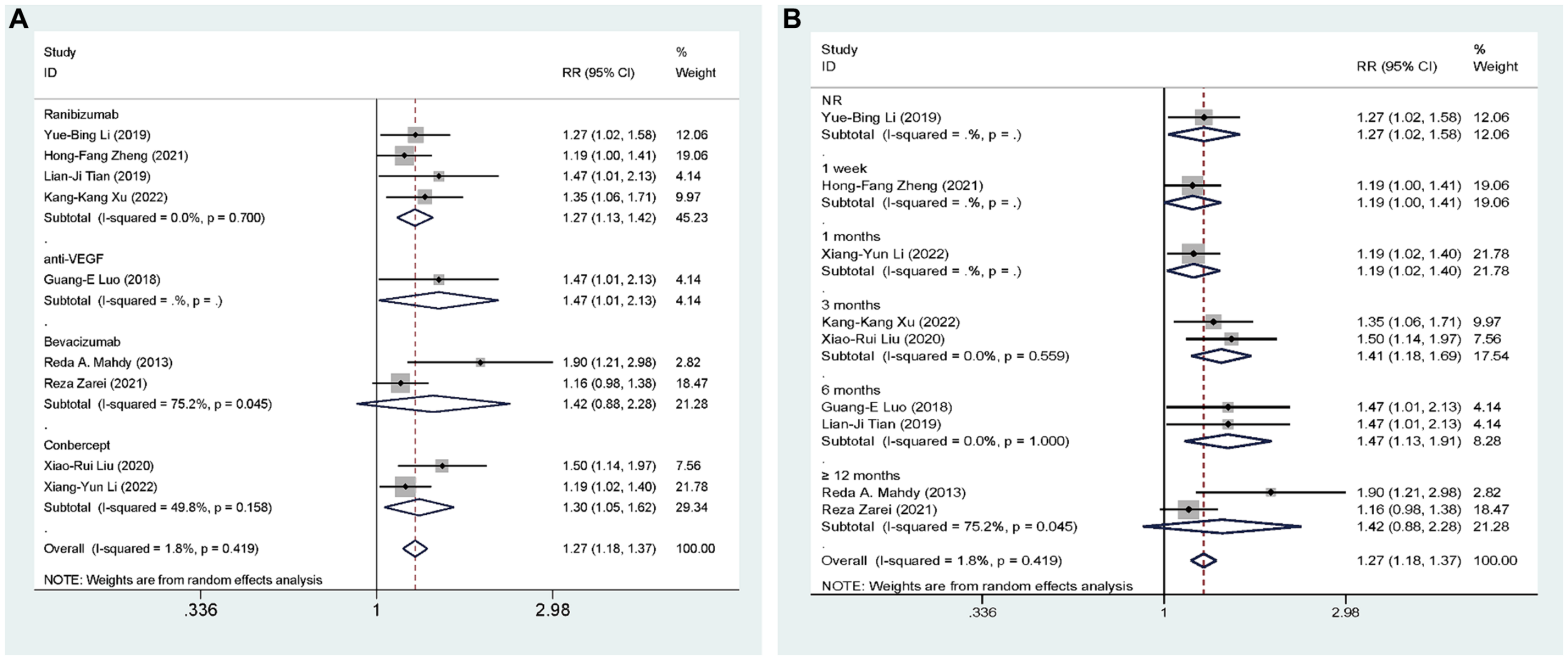
AGVI alone vs. AGVI combined with anti-VEGF drugs: **(A)** Effective rate: subgroup analysis based on anti-VEGF drugs; **(B)** Effective rate: subgroup analysis based on follow-up time.

Nine studies (27, 28, 31, 35–40), including 628 eyes, compared the overall efficacy of AGVI combined with anti-VEGF drugs versus AGVI alone in patients with NVG. The aggregated findings demonstrated a superior overall efficacy of AGVI combined with anti-VEGF drugs compared to AGVI alone [RR = 1.27, 95% CI (1.18, 1.37), *p* < 0.001]. The summarized findings indicated that both ranibizumab group and conbercept group all demonstrated a significant positive impact on enhancing effective rates. ([RR = 1.27, 95% CI (1.13, 1.42), *p* < 0.001] and [RR = 1.30, 95% CI (1.05, 1.62), *p* = 0.017] respectively). However, the outcomes of bevacizumab group did not reach statistical significance (*p* = 0.149) (Figure 5A). Subgroup analysis based on follow-up time revealed that the efficacy of anti-VEGF drugs combined with AGVI was superior to AGVI alone at 3 months and 6 months post-operation ([RR = 1.41, 95% CI (1.18, 1.69), *p* < 0.001] and [RR = 1.47, 95% CI (1.13, 1.91), *p* = 0.004] respectively). Nevertheless, there was no significant difference in efficacy between the two groups beyond 12 months post-operation [RR = 1.42, 95% CI (0.88, 2.28), *p* = 0.149] (Figure 5B).

#### 3.4.7 Incidence of postoperative hyphema

**Figure 6:**
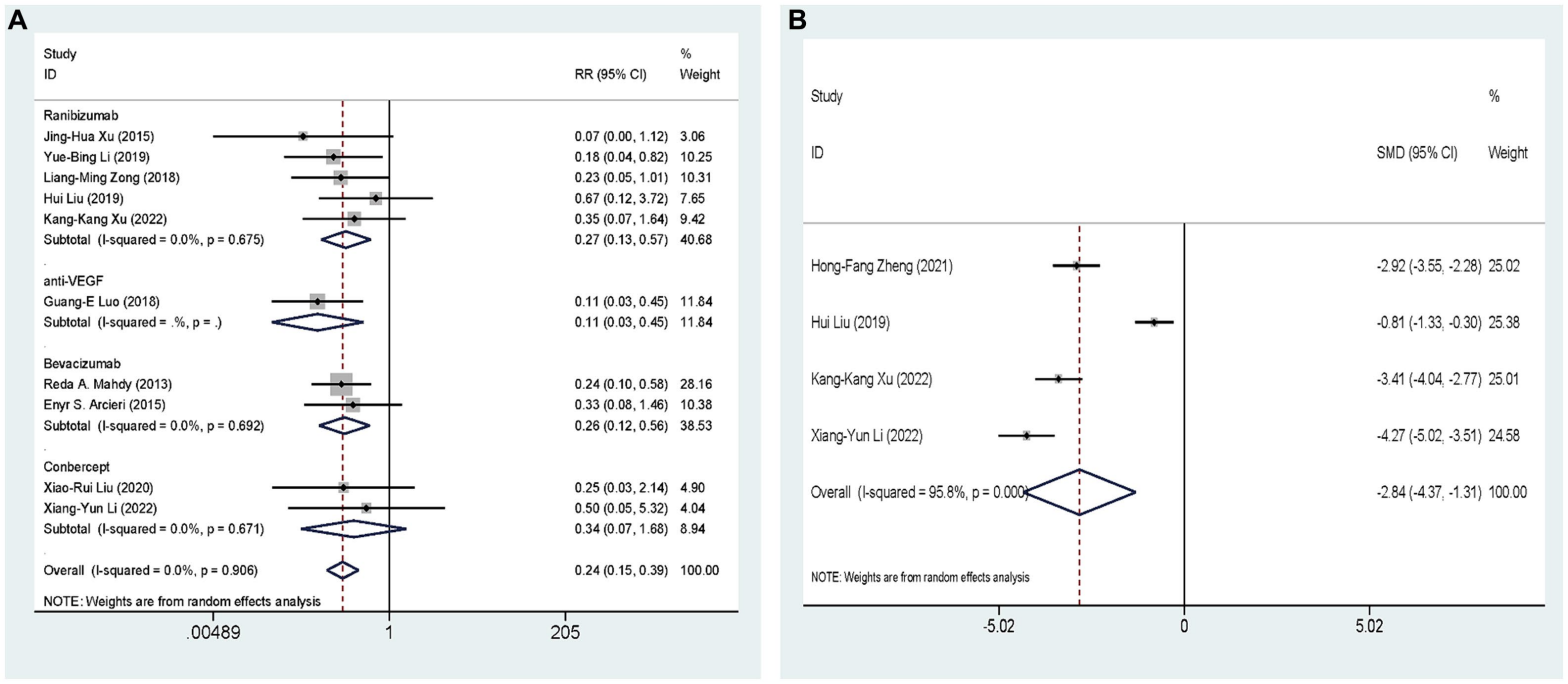
AGVI alone vs. AGVI combined with anti-VEGF drugs: **(A)** Incidence of postoperative hyphema: subgroup analysis based on anti-VEGF drugs; **(B)** Aqueous humor VEGF levels.

Ten studies (27, 29–32, 34, 35, 37, 38, 40), including 641 eyes, compared the incidence of postoperative hyphema of AGVI combined with anti-VEGF drugs versus AGVI alone in patients with NVG. The pooled results showed that compared to AGVI alone, the combination of AGVI with anti-VEGF drugs effectively reduces the probability of postoperative hyphema [RR = 0.24, 95% CI (0.15, 0.39), *p* < 0.001]. The results of subgroup analysis indicated that the ranibizumab group and bevacizumab group all demonstrated superior efficacy in reducing the incidence of postoperative hyphema ([RR = 0.27, 95% CI (0.13, 0.57), *p* = 0.001] and [RR = 0.26, 95% CI (0.12, 0.56), *p* = 0.001]). Conversely, the outcomes of conbercept group did not reach statistical significance (*p* = 0.186) (Figure 6A).

#### 3.4.8 Aqueous humor VEGF levels

Four RCTs (34, 35, 38, 39), comprising 326 eyes, investigated the effects of combined AGVI with anti-VEGF drugs versus AGVI alone on aqueous humor VEGF levels. Due to the different units for measuring aqueous humor VEGF levels in the included literature, we conducted the analysis using SMD. The pooled results showed that compared to AGVI alone, the combination of AGVI with anti-VEGF drugs had a better effect on reducing aqueous humor VEGF levels [SMD = −2.84, 95% CI (−4.37, −1.31), *p* < 0.001] (Figure 6B).

#### 3.4.9 Postoperative antiglaucoma medication requirements

**Figure 7:**
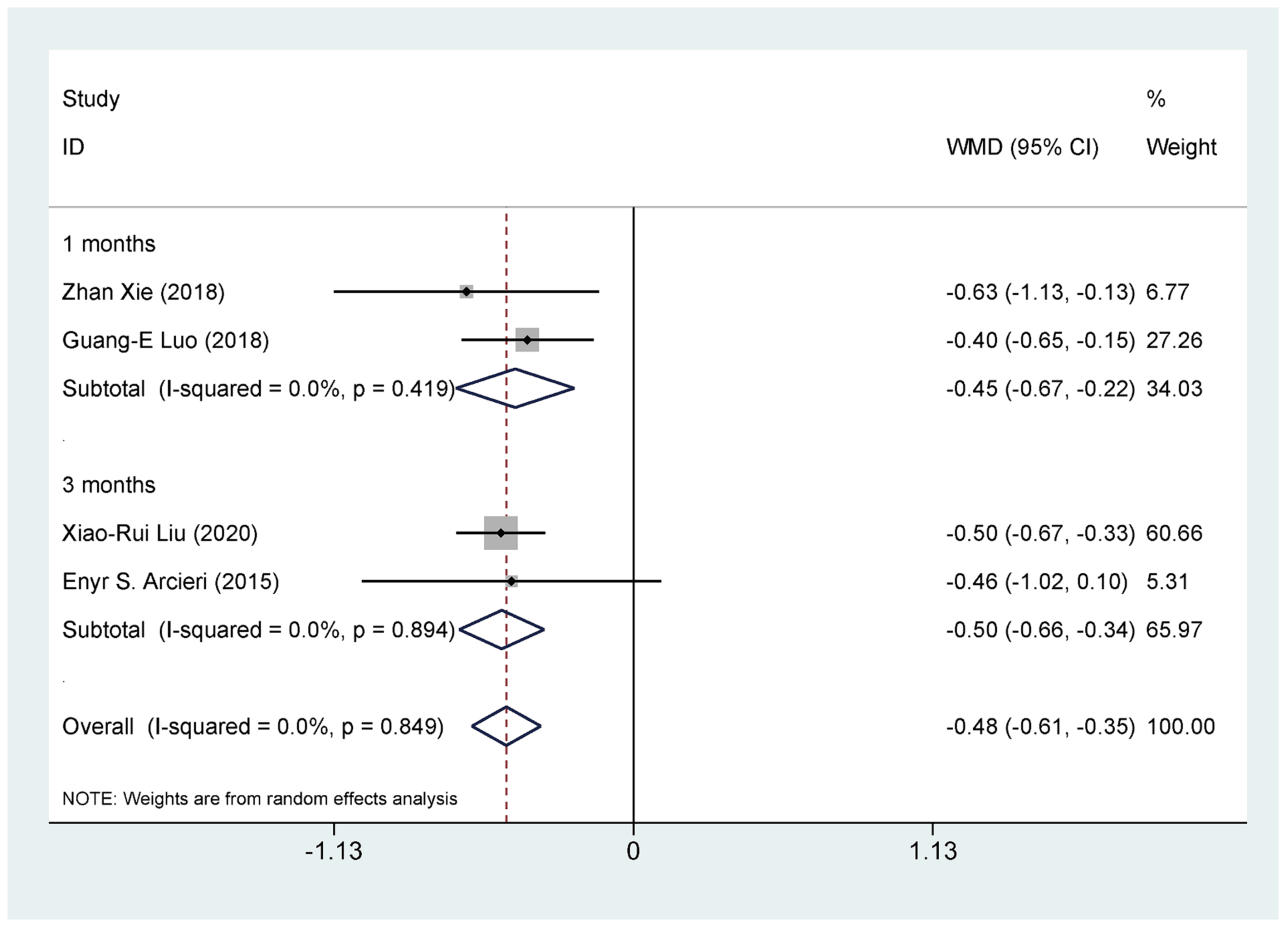
AGVI alone vs. AGVI combined with anti-VEGF drugs: postoperative antiglaucoma medication requirements.

Four studies (29, 33, 37, 40), involving 238 eyes, compared the impact of AGVI combined with anti-VEGF drugs versus AGVI alone on postoperative glaucoma medication use in NVG patients. The pooled results showed that compared to AGVI alone, AGVI combined with anti-VEGF drugs effectively reduced the postoperative use of antiglaucoma medications [WMD = −0.48, 95% CI (−0.61, −0.35), *p* < 0.001] (Figure 7). Subgroup analysis based on follow-up time revealed that the use of anti-glaucoma medications was significantly lower with the combination of anti-VEGF drugs and AGVI than with AGVI alone at both 1 month and 3 months post-operation ([WMD = −0.45, 95% CI (−0.67, −0.22), *p* < 0.001] and [WMD = −0.50, 95% CI (−0.66, −0.34), *p* < 0.001] respectively) (Figure 7).

### 3.5 Sensitivity analysis

We conducted a sensitivity analysis to assess the impact of individual studies on the overall results of comparing the treatment outcomes for NVG using anti-VEGF drugs combined with AGVI versus AGVI alone. The findings revealed that no single study significantly impacted the final results, suggesting the robustness and stability of the study’s findings (Figure 7).

### 3.6 Publication bias

**Figure 8:**
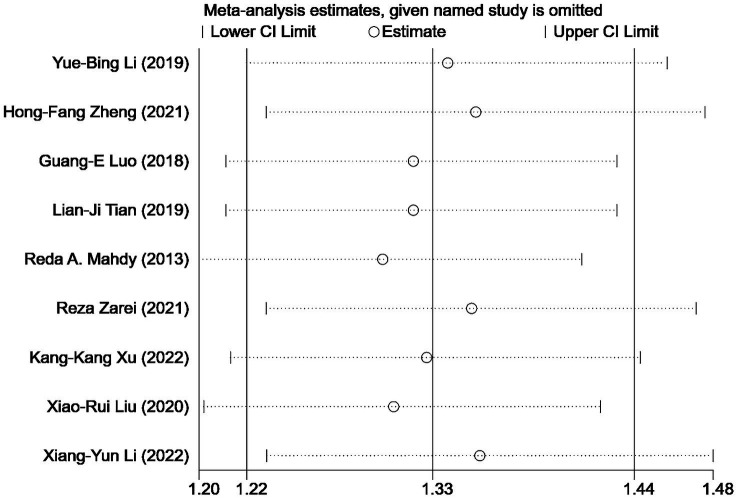
Sensitivity analysis: effective rate of AGVI alone vs. AGVI combined with anti-VEGF drugs.

For outcome metrics that include 10 or more studies, we visually inspected funnel plots to explore the potential for publication bias, the funnel plots appeared slightly asymmetrical. To go further with the exploration, we used Egger’s test. Egger’s test yielded a *p* value of 0.835, suggesting that publication bias is unlikely to have significantly influenced the results of this study (Figure 8).

## 4 Discussion

### 4.1 Summary of results

This study analyzed the effectiveness and safety of combined anti-VEGF drugs and AGVI treatment for NVG, examining bevacizumab, ranibizumab, and conbercept. Results from the study demonstrated that combining anti-VEGF medications with AGVI resulted in superior outcomes compared to AGVI alone, including reduced postoperative IOP, decreased incidence of postoperative hyphema, diminished use of postoperative anti-glaucoma medications, and decreased intraocular VEGF levels. In terms of reducing intraocular pressure, both at 1 week and 1 month postoperatively, combined therapy achieved better results and could reduce postoperative medication use, consistent with the meta-analysis results by Lin et al. (41). Simultaneously, the combined therapy group exhibited a higher success rate and fewer postoperative adverse reactions, consistent with the meta-analysis findings by Chen and Mu (42) and Hwang and Lee (43). More importantly, through our research, we found that the combined therapy group had better long-term control of IOP postoperatively compared to AGVI alone. To this end, we analyzed IOP data from follow-up visits at 3 months, 6 months, and beyond 12 months postoperatively. Regarding safety, we found that the combination of anti-VEGF drugs with AGVI reduced the incidence of postoperative hyphema compared to AGVI alone. This finding is consistent with Zhou et al. (44) meta-analysis on the treatment of NVG with bevacizumab combined with AGVI.

To further explore the therapeutic effects of combining anti-VEGF drugs with AGVI on NVG, subgroup analyses were conducted on the anti-VEGF medications, including the ranibizumab, bevacizumab, and conbercept groups. Studies that did not specify the type of anti-VEGF medication used were excluded from the subgroup analyses. In the analysis of IOP results at 1 week postoperatively, all three subgroups exhibited statistically significant differences. Besides, ranibizumab group and conbercept group all demonstrated superior efficacy in reducing intraocular pressure at 1 month postoperatively compared to AGVI alone. At 3 months postoperatively, both the bevacizumab and ranibizumab groups demonstrated significant differences compared to AGVI alone. Regarding the IOP results at 6 months postoperatively, the bevacizumab group did not exhibit statistically significant differences, while the ranibizumab group showed significant differences compared to AGVI alone. However, the conbercept group did not demonstrate significant differences in reducing postoperative hyphema. Subgroup analysis based on follow-up time revealed that the efficacy of anti-VEGF drugs combined with AGVI was superior to that of AGVI alone at 3 months and 6 months post-operation. However, there was no significant difference in efficacy between the two groups beyond 12 months post-operation. Additionally, the combination group significantly reduced the use of anti-glaucoma medications at 1 month and 3 months post-operation.

### 4.2 Discussion of anti-VEGF combined with AGVI vs. AGVI alone

NVG is a severe secondary glaucoma. Although endoscopic cyclophotocoagulation, cyclocryotherapy, simple trabeculectomy, and traditional filtering surgery have certain therapeutic effects, the postoperative conditions of intraocular hypertension, retinal detachment, loss of vision, and atrophy of the eyeball cannot be ignored (45). AVGI modulates the flow of aqueous humor by positioning a tube between the conjunctiva and sclera, theoretically, the incorporation of a restrictive valve-like mechanism within the AGVI confers substantial advantages in controlling early postoperative IOP and reducing the risk of hypotony in contrast to trabeculectomy (46). Drainage valve implantation is also considered the gold standard for treating NVG (47). A meta-analysis by Tan et al. (48) found that AGVI yielded similar outcomes to trabeculectomy, but with a significantly reduced incidence of postoperative complications. However, in a retrospective study, Netland (49) reported a success rate of 73.1% at 1 year, 61.9% at 2 years, and only 20.6% at 5 years, and considered NVG a high risk factor of AGV implantation failure.

Intraocular anti-VEGF drugs are increasingly utilized as adjunctive therapy for refractory NVG to enhance the outcomes of glaucoma surgery in high-risk patients (50). VEGF is a potent angiogenic stimulator, facilitating multiple stages of angiogenesis such as proliferation, migration, proteolytic activity, and capillary tube formation, it plays a crucial role in both normal and pathological angiogenesis (51). In patients with NVG, the concentration of VEGF is elevated in the aqueous humor, more importantly, an abundance of VEGF enters the anterior chamber of the eye from the posterior pole, initiating neovascularization (NV) primarily from the capillaries of the minor and major arterial rings of the iris before extending to the anterior chamber angle, it also leads to disruption of the blood-retinal barrier (52, 53). The currently available anti-VEGF inhibitors, including bevacizumab and ranibizumab, have proven to be effective suppressing anterior segment neovascularization and lowering IOP (4). In order to further investigate the effectiveness of anti-VEGF medications, this meta-analysis included a subgroup analysis of three distinct anti-VEGF drugs. According to the results of subgroup analysis, ranibizumab achieved favorable outcomes. Compared to AGVI alone, combination therapy with ranibizumab can lower IOP in both short-term and long-term postoperative periods, reduce postoperative medication usage, and decrease the incidence of hyphema. This may be attributed to ranibizumab’s function as an antagonist of vascular endothelial growth factor-A (VEGF-A), allowing it to bind with high affinity to various VEGF-A isoforms (54). The binding of ranibizumab to VEGF-A inhibits the interaction between VEGF-A and its receptors (VEGFR1 and VEGFR2) on endothelial cell surfaces, thereby decreasing endothelial cell proliferation, vascular leakage, and angiogenesis (4). Ranibizumab is a monoclonal antibody fragment produced by the gram-negative bacterium *Escherichia coli*, with a molecular weight of 48 kD, the rationale for developing Ranibizumab as a fragment antigen-binding (Fab) is based on the hypothesis that its small size enables better tissue penetration through all retinal layers (55). Each molecule of ranibizumab possesses only one binding site for VEGF, allowing each VEGF dimer to be bound by two ranibizumab molecules (56). A study found that at clinically significant doses, bevacizumab (0.25 mg/mL) and ranibizumab (0.125 mg/mL) could completely neutralize vascular endothelial growth factor within 6 h, when diluted, bevacizumab lost its inhibitory effect at a concentration of 975 ng/mL, while ranibizumab neutralized vascular endothelial growth factor at a concentration of 120 ng/mL (57). Although the subgroup analysis results found that bevacizumab’s performance in lowering IOP at 6 months postoperatively was suboptimal, it still reduced the occurrence of hyphema. Bevacizumab as a full-length humanized, can recombinant monoclonal IgG antibody that inactivates all VEGF isoforms (58). Ha et al. (59) found in a retrospective study that among 26 patients NVG who received intravitreal injections of bevacizumab, IOP significantly decreased 1 week later, but the effect was not significant at the one-year follow-up. Ghanem et al. (60) found that 1 week after intravitreal injection of bevacizumab, significant regression of iris neovascularization could be observed in eyes with NVG. The subgroup analysis of conbercept revealed that, when compared to AGVI alone, combination therapy with conbercept can effectively decrease IOP in both short-term and long-term postoperative periods, as well as reduce the need for postoperative medication. However, it appeared to have no effect on reducing the incidence of hyphema. Placental growth factor (PlGF) primarily functions as a pro-angiogenic growth factor, exhibiting upregulation specifically in pathological conditions and conbercept can bind to dual targets (VEGF and PIGF) for antiangiogenic therapy (61). Xu et al.’s study found that intravitreal injection of conbercept can reduce the levels of IL-4, IL-22, Ang-2, PlGF, and VEGF-A in the aqueous humor (62). Subgroup analysis based on follow-up time revealed that the combination of anti-VEGF drugs and AGVI did not improve efficacy beyond 12 months post-operation. Additionally, a study by Rittiphairoj et al. (63). found no evidence that VEGF drugs can maintain long-term efficacy as an adjunct therapy. This could be because after regression of neovascularization, the iridocorneal angle appears open on gonioscopy, however, ghost vessels, which are transparent and tend to form synechiae, can lead to subsequent angle closure, additionally, VEGF cannot work on the fibrovascular membrane that closes the iridocorneal angle (64). This also may be due to the regression of iris neovascularization, which can persist for 8–10 weeks after intraocular injection but typically returns to its previous condition within 6 months. Consequently, this treatment plays a limited and temporary role in managing NVG (24). More rigorous evidence is still needed to confirm that anti-VEGF drugs can serve as an effective long-term adjunct to AGVI. According to previous studies, PRP can effectively improve retinal ischemic conditions and reduce the release of VEGF, thus preventing the development of NVG, additionally, PRP facilitates the control of IOP and enhances the long-term outcomes of surgery (65). None of the studies included in this research explicitly mentioned not using PRP, therefore, we were unable to conduct a deeper analysis of the role of PRP through subgroup analysis or other methods.

During the course of this study, we found that the majority of cases were from the same region. For that, we explored the possible reasons for this phenomenon by searching relevant literature. In a meta-analysis by Tham et al. (66), it was found that due to the relatively large population of Asians, over half (53.4%) of global primary open-angle glaucoma (POAG) cases occurred in Asia, in addition, primary angle-closure glaucoma (PACG) is the predominant type of glaucoma in the Asian population (1.1%), with over three-quarters (76.7%) of global PACG cases occurring in Asia. According to Song et al.’s study (67), the number of patients with secondary glaucoma in China increased from 340,000 (95%CI = 0.23–0.53) to 760,000 (95%CI = 0.51–1.17) by 2015. A retrospective study from a tertiary center in China revealed that patients with NVG constituted approximately 5.8% of all glaucoma patients in China (68). More importantly, the prevalence of glaucoma is positively correlated with advanced age, as China stands as the largest developing country, its population is rapidly aging, which results in a significant burden of glaucoma (69). Based on the aforementioned factors, this may explain why a large number of cases come from the same region.

### 4.3 Strength and limitations

This study has the following advantages: First, it includes an analysis of the current mainstream anti-VEGF drugs, which is more comprehensive and complete compared to analyzing a single anti-VEGF drug. Secondly, the paper conducts a subgroup analysis of three distinct anti-VEGF drug categories, aiming to aid clinicians in the selection of appropriate treatments. Thirdly, the outcome indicators included in this study are relatively comprehensive, covering both short-term and long-term postoperative IOP, BCVA, Incidence of postoperative hyphema, efficacy, Postoperative antiglaucoma medication requirements and aqueous humor VEGF levels. More importantly, compared to previous meta-analyses on the subject, this study exclusively included RCTs, excluding other types of research.

This study also presents certain limitations. Firstly, it is restricted to analyses of clinical practices and published studies; thus, it does not encompass research on anti-VEGF drugs currently under investigation or unpublished, including faricimab, nesvacumab, and squalamine. Secondly, the study did not exclude patients with NVG who may also suffer from other retinal conditions, such as diabetic retinopathy. Thirdly, despite the adoption of a multi-person collaborative assessment method, the bias risk assessment tool used in this systematic review still harbors unavoidable subjectivity, which may affect the final evaluation of the literature quality. Finally, as most of the included studies originate from the same region, their findings may have limited global applicability. Future research from diverse regions is necessary to validate the conclusions of this study.

## 5 Conclusion

In conclusion, compared to AGVI alone, the combination of AGVI with anti-VEGF drugs has a good effect in controlling postoperative intraocular pressure at different times, increasing the effective rate, reducing aqueous VEGF levels, decreasing the use of postoperative antiglaucoma drugs, and also reducing the occurrence of postoperative anterior chamber hemorrhage. Additionally, this study also found that the efficacy of ranibizumab appears to be more stable. In the future, it will be necessary to conduct multicenter, randomized, double-blind, large-sample, rigorously designed clinical trials with long-term follow-up to confirm the conclusions of this study.

## Data availability statement

The original contributions presented in the study are included in the article/Supplementary material, further inquiries can be directed to the corresponding author.

## Author contributions

C-ZH: Writing – original draft, Writing – review & editing. S-JL: Data curation, Methodology, Writing – review & editing. Z-JZ: Software, Writing – original draft. J-QL: Writing – review & editing. QQ: Writing – review & editing. F-LX: Writing – review & editing. YH: Writing – review & editing.
